# Making Insulin and Staying Out of Autoimmune Trouble: The Beta-Cell Conundrum

**DOI:** 10.3389/fimmu.2021.639682

**Published:** 2021-03-29

**Authors:** Alexia Carré, Roberto Mallone

**Affiliations:** ^1^ Université de Paris, Institut Cochin, CNRS, INSERM, Paris, France; ^2^ Assistance Publique Hôpitaux de Paris, Service de Diabétologie et Immunologie Clinique, Cochin Hospital, Paris, France

**Keywords:** antigen presentation, antigen processing, autophagy, crinophagy, insulin granule, MHC class I, neo-epitopes, signal peptide

## Abstract

Autoimmune type 1 diabetes (T1D) results from the intricate crosstalk of various immune cell types. CD8+ T cells dominate the pro-inflammatory milieu of islet infiltration (insulitis), and are considered as key effectors of beta-cell destruction, through the recognition of MHC Class I-peptide complexes. The pathways generating MHC Class I-restricted antigens in beta cells are poorly documented. Given their specialized insulin secretory function, the associated granule processing and degradation pathways, basal endoplasmic reticulum stress and susceptibility to additional stressors, alternative antigen processing and presentation (APP) pathways are likely to play a significant role in the generation of the beta-cell immunopeptidome. As direct evidence is missing, we here intersect the specificities of beta-cell function and the literature about APP in other cellular models to generate some hypotheses on APPs relevant to beta cells. We further elaborate on the potential role of these pathways in T1D pathogenesis, based on the current knowledge of antigens presented by beta cells. A better understanding of these pathways may pinpoint novel mechanisms amenable to therapeutic targeting to modulate the immunogenicity of beta cells.

## Introduction

**Figure 1:**
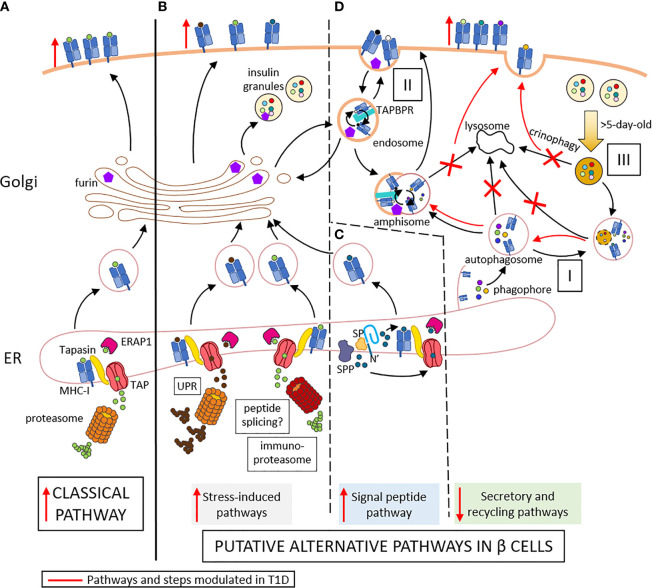
Conventional **(A)** and putative alternative APP pathways **(B–D)** in beta cells. **(A)** In conventional APP pathways, misfolded proteins are degraded by the proteasome into peptides that are transported through TAP into the ER lumen. Here, they are further trimmed by ERAP1 and loaded onto MHC-I molecules that are associated to TAP *via* tapasin. The peptide-MHC-I complexes are exported to the Golgi and then to the cell surface. **(B)** Under inflammatory conditions, ER stress induces the UPR, which increases protein degradation and antigen presentation. The immuno-proteasome is also induced, further increasing APP and possibly resulting in alternative peptide splicing events and neo-epitopes generated. **(C)** Signal peptides are docked within the ER membrane. While the protein translation continues, the signal peptide is cleaved by the signal peptidase (SP) and further trimmed by the signal peptide peptidase (SPP). The N-terminal cleavage products of the signal peptide are released in the cytosol and can re-enter the ER through TAP for antigen presentation. The C-terminal cleavage products remaining in the ER can be loaded onto MHC-I molecules in a TAP- and proteasome-independent manner. **(D)** I. Cellular components are engulfed by nascent phagophore at the ER membrane, thus forming autophagosome, likely containing MHC-I molecules. II. Endocytosis of cell surface component forms endosomes, which can contain both MHC-I molecules and TAPBR promoting peptide exchange within endosomes. These vesicles can be directed to the Golgi apparatus, where peptides (free or released from MHC-I) can be trimmed by Golgi enzymes such as furins prior to loading onto MHC-I molecules. Autophagosomes, if not directly fused to lysosomes for degradation of their content, can fuse with endosome to form amphisomes, thus opening another peptide exchange pathway. These vesicles can subsequently be directed to lysosomes for degradation. III. Old insulin granules can be degraded by crinophagy. T1D induces changes (in red) characterized by enhanced ER stress **(B)**, increased insulin production and signal peptide processing **(C)**, and altered vesicle trafficking **(D)**, thus enhancing surface exposure of MHC-I complexes. Red marks indicate changes occuring in T1D: vertical arrows represent the increase or decrease of a step or pathway; bended arrows represent favored events; crosses represent blocked events. Some cellular components were modified from Servier Medical Art (smart.servier.com).

Type 1 diabetes (T1D) is an autoimmune disease characterized by the destruction of insulin-producing beta cells. It stems from a complex interplay of innate and adaptive immune cells. CD8+ T cells dominate the immune infiltration of islets and play a prominent role as final effectors of beta-cell loss (1). There is also growing evidence supporting the idea that beta-cell dysfunction is another key driver of T1D pathogenesis (2). The heterogeneity of pancreas histopathology between T1D donors and even across islets from the same pancreas, both in terms of immune infiltrates and residual beta cells, have led to the definition of age-related endotypes (3), in which the component of beta-cell dysfunction may be dominant in adult-onset cases (4, 5). Effector CD8+ T cells recognize MHC Class I (MHC-I)-peptide complexes at the surface of beta cells. The conventional MHC-I antigen processing and presentation (APP) machinery is a multi-step process (**Figure 1A**) where *i)* cytosolic proteins of microbial or self-origin are degraded into peptides in a proteasome-mediated manner; *ii)* the resulting peptides are transported into the endoplasmic reticulum (ER) *via* the transporter associated with antigen processing (TAP). Here, *iii)* they are further trimmed by ER aminopeptidase (ERAP)1 prior to *iv)* loading on nascently-formed MHC-I molecules that are associated to TAP through tapasin; *v)* the stable peptide-MHC-I complexes are finally translocated to the Golgi complex and to the cell surface (6). This review addresses the specificities of the direct alternative MHC-I APP (aAPP) machinery within beta cells and its potential role in T1D pathogenesis (**Figures 1B–D**). It therefore focuses on the CD8+ T-cell responses that are triggered by MHC-I APP, notwithstanding the role of other cell types, and particularly of MHC-I and MHC-II APP by professional antigen-presenting cells (APCs) for the priming of naïve T cells. Since the beta-cell APP pathways are poorly if at all documented, we here provide a hypothesis-generating review that bridges the separate bodies of knowledge available for MHC-I APP and beta-cell biology.

## Secreting Insulin: The Achilles’ Heel of Beta Cells?

Like other endocrine cells, beta cells are hormone-secreting cells organized in glands that are highly vascularized, allowing the secretion of their products directly into the bloodstream. These features may influence the APP pathways used by these cells (2). Indeed, beta cells are equipped to sense changes in blood glucose levels and to respond by releasing appropriate amounts of insulin. For this purpose, they need to constantly adapt their secretory response to changes in metabolic and nutritional state. This adaptation is critical compared to that of other tissues because beta cells are long-lived and virtually non-proliferating, hence they can only modulate their function but not their cell numbers. Beta cells accommodate these metabolic variations by intensifying the synthesis of insulin and its precursor proinsulin. Under these intense bio-synthetic rates, misfolded proteins are more likely to accumulate within the ER, activating the unfolded protein response (UPR) (7). The UPR is a natural adaptive response that maintains cellular homeostasis by fulfilling three main tasks: to decrease the protein translation rate, to enhance the synthesis of protein-folding chaperones, and to increase the degradation of misfolded proteins into peptides through the proteasome (7). While part of the free cytosolic peptides generated by this process is further hydrolyzed to single amino acids, another portion is transported to the ER *via* TAP for the loading onto MHC-I molecules, thus shaping the catalog of MHC-I-bound peptides (immunopeptidome) presented at the beta-cell surface (**Figure 1B**). Beta cells rely on a well-balanced UPR to ensure their function and survival (8). Thus, beta cells are constantly functioning on the edge between physiological and pathological UPR and are exquisitely sensitive to additional stressors that can flip this delicate balance and lead to a compensatory maladaptive UPR further increasing ER stress prior to apoptosis (9, 10). Metabolic overload can act as an accelerator but is unlikely to drive this UPR transition on its own, as such overload is more common in type 2 diabetes and yet does not usually trigger islet autoimmunity. The environmental factors at play are likely multiple, but their identity and interaction with a susceptible genetic background remain ill-defined, with the possible exception of enteroviral infections (11). Considering the link between the UPR and APP through increased proteasomal degradation of misfolded proteins, the shift from physiological to pathological UPR may modulate the immunopeptidome and the beta-cell visibility to the immune system.

Under physiological conditions, the beta cells maintain consistent intracellular stores of insulin granules to allow their rapid exocytosis when the glycemia rises. While insulin granules are found by thousands in individual beta cells (12), not all are secreted. Indeed, the remarkable functional plasticity of beta cells includes recycling pathways to dispose of old (>5 days) or excess secretory granules. Commonly, insulin granules are degraded through macro-autophagy, by which they are engulfed by autophagosomes prior to degradation in lysosomes. Another endocrine-specific recycling pathway is a specialized macro-autophagy process named crinophagy, by which insulin granules fuse directly with lysosomes, thus generating so-called crinosomes (**Figure 1D**). While the role of these different types of autophagy has been extensively studied in type 2 diabetes, investigations in T1D are scanty. One recent study (13) reported defective macro-autophagy and diminished crinophagy in the beta cells of non-obese diabetic (NOD) mice compared to non-diabetic NOD littermates and non-obese resistant (NOR) mice. These conclusions were confirmed in T1D pancreas specimens and were based on the lack of co-localization of the lysosomal marker Lamp1 with either the granule marker proinsulin or the autophagosome marker LC3, thus suggesting a defect in the late stage of autophagy, when granules/autophagosomes fuse with lysosomes. An alternative explanation is that this reduced crinophagy may simply reflect a limited accumulation of old granules due to a compensatory higher insulin exocytosis rate in remaining beta cells. Interestingly, this study also reported a parallel increase of autophagosomes in insulin-positive cells of pancreata from autoantibody-positive non-diabetic donors in comparison to both control and T1D donors, whose autophagosome content was instead similar. As autophagy helps to prevent oxidative damage and promote cell survival, we can hypothesize that the beta-cell stress imposed by the inflammatory milieu of insulitis triggers an increase in their autophagy rate (14), prior to, and likely independently of, overt hyperglycemia. Interestingly, lysosomal autophagy can also degrade mitochondria (15). This process, known as mitophagy, is also part of stress responses (16), and is regulated by CLEC16A, whose genetic locus encodes variants associated with T1D risk (17). Pancreatic CLEC16A knockout mice harbor a defect in both mitochondrial and granule turnover (16), pointing to an intersection between the mitophagy and crinophagy pathway. It is thus possible that the altered granule disposal observed in stressed beta cells may also reflect overloading of this pathway by mitochondrial substrates. Its modulation by the *CLEC16A* genetic background may exacerbate the effect of stress on aAPPs. This feature also emphasizes the current view of lysosomes as dynamic structures that interact with different intracellular organelles and are continuously consumed and re-formed rather than just being end-stage degradation hubs (15).

Given this notion of impaired autophagy in T1D (13), what is the fate of the autophagosomes and insulin granules that are not degraded by lysosomes? In non-professional APCs, surface peptide-loaded MHC-I molecules seem to be recycled by clathrin-independent mechanisms, forming endosomes (18, 19). Whether and how these endosomes are degraded by lysosomes or targeted toward an early or late recycling pathway is unclear. Nonetheless, peptide exchange for MHC-I loading is likely to occur within the endosome. The TAP binding protein related (TAPBPR) chaperone is currently held as the main candidate for this role (20), and its preference for a slightly acidic to neutral pH is in the range of that found in endosomes. Another possibility is the encounter between endosomes and autophagosomes prior to recycling (21), forming so-called amphisomes (**Figure 1D**). Hence, the autophagosomes that are not degraded by lysosomes may be more likely to fuse with endosomes and gain access to the cell surface. Considering that the autophagosome membrane is thought to derive from the ER and can thus harbor MHC-I molecules amenable to peptide loading (22), this process may provide an aAPP pathway. Similarly, insulin granules that are not degraded by lysosomes could indirectly participate in APP through engulfment by autophagosomes. Collectively, these data suggest that alternative autophagy-derived pathways for MHC-I loading could be operational in beta cells and participate in the recycling processes needed for disposing of insulin granules. While these pathways are active in resting beta cells, they may be further enriched in T1D as autophagy is impaired, possibly providing novel disease-enhanced pathways.

**Figure 2:**
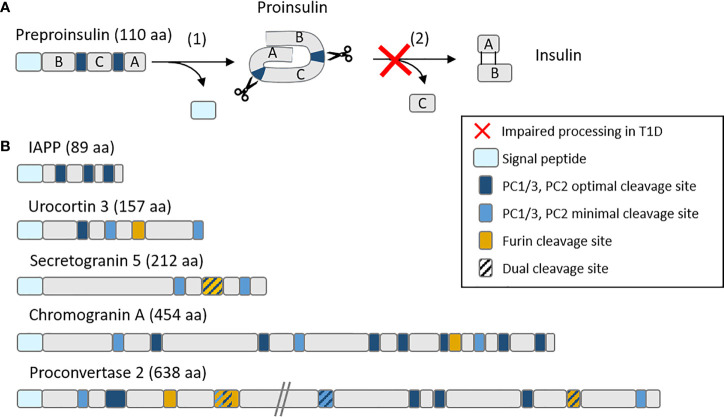
Schematic representation of **(A)** the insulin maturation process and **(B)** mapping of proconvertase cleavage sites within insulin granule proteins. **(A)** Insulin is translated as a preprohormone comprising a signal peptide (light blue), followed by the B chain, C peptide and A chain. The first processing step (1) consists in the cleavage of the signal peptide, followed by (2) the cleavage by proconvertases of the C peptide and formation of disulfide bonds between the A and B chain. In T1D, this second step is often impaired, leading to proinsulin accumulation. **(B)** PC1/3 and PC2 optimal (RR, KR) and minimal (KK, RK) cleavage sites along with furin cleavage sites (R-X-X-R) (where X is any amino acid) are mapped on major insulin granules proteins. aa, amino acids.

Besides insulin, secretory granules contain other proteins that we reported to dominate the beta-cell-specific immunopeptidome targeted by islet autoimmunity. First, a large fraction of MHC-I-bound peptides recovered from a human beta-cell line and primary islets originate from these granule proteins (23), which is not unexpected given the abundance and fast turnover of these proteins in beta cells (24). Second and most important however, these granule-derived peptides were also prominent targets recognized by circulating CD8+ T cells (23, 25). More recently, we found that H2-Kd-restricted peptides derived from the murine orthologues of some of these proteins, namely secretogranin-5, urocortin-3 and proconvertase-2, are also recognized by islet-infiltrating CD8+ T cells in prediabetic NOD mice (25). Moreover, CD8+ T cells recognizing these peptides were diabetogenic upon in-vivo transfer into NOD/*scid* recipients (25). Besides their localization in granules, it is noteworthy that these novel antigens (together with chromogranin A and others) (26) share several other features with insulin. First, they are all soluble proteins that are released along with insulin during granule exocytosis, a feature that could endow beta cells with a unique capability of sensitizing T cells at distance following APP of these proteins by extra-pancreatic APCs (2, 27). Second, they are all produced as precursors (pro-proteins) and subsequently undergo intermediate processing, first in the ER to cleave the signal peptide (see below), and then in immature granules through proconvertases, carboxypeptidase E (CPE) and furins that lead to their bioactive products (**Figure 2**). Intriguingly, reduced levels of proconvertases and CPE along with impaired proconvertase activity and proinsulin processing in beta cells has been repeatedly reported for T1D, even before clinical onset (4, 28–32). These enzymatic defects could lead to increase protein misfolding, which might feed the proteasomal recycling pathway and further fuel the APP machinery. It is also possible that granule protein byproducts may accumulate in the ER, the Golgi or the cytosol and thus become accessible for processing by other enzymes and for loading onto MHC-I molecules, either directly, through retrograde transport to the ER, or indirectly, *via* TAP. Of further note, proconvertases themselves as well as (pro)cathepsins are also synthesized as inactive precursors and subsequently activated, often through auto-enzymatic reactions. We can thus speculate that ER stress and impaired proconvertase activity may also negatively impact these processes since their earliest steps of ER export and self-activation.

An unsolved conundrum is how the T-cell targeting of granule antigens that are mostly shared with other endocrine cells such as alpha cells leads to an autoimmune response that is exquisitely beta-cell-specific. This question is even more important in light of recent reports suggesting that MHC–I upregulation is more prominent in alpha cells, even before clinical T1D (33). Protective factors at play in alpha cells may include their lower biosynthetic rate, the narrower dynamic range of their secretory response (34), their lower susceptibility to ER stress-induced apoptosis (35), and their higher expression of non-classical, inhibitory MHC-I molecules, i.e. human leukocyte antigen (HLA)-E (36). Moreover, autophagy seems less important for the maintenance of alpha-cell metabolism (37) than it is for beta cells (38), possibly making autophagy-related APP pathways less active.

## Neo-Epitope Generation in Beta Cells

The large stores of insulin granules and the functional adaptability to metabolic changes of beta cells point to a remarkably active translation machinery, which enhances the odds of neo-epitope generation. Neo-epitopes are unconventional peptides such as post-translational modified sequences, defective ribosomal products (DRiPs), alternative mRNA splicing products and proteasomal peptide splicing products (i.e. resulting from the fusion of two non-adjacent regions of a protein). We reported that some of these products contribute to the MHC-I peptidome of beta cells (23, 25, 26). Very little is however known about how such peptides are generated and whether the required pathways are active in beta cells and/or professional APCs. In spite of their tissue specificity, the overall quantitative contribution of neo-epitopes to the beta-cell immunopeptidome appears to be minor, as only a limited number of MHC-I-restricted neo-epitopes has been reported to date (26). A possible scenario is that these neo-epitopes might be symptomatic of early beta-cell dysfunction, e.g. altered granule bio-synthesis/disposal, and become visible to T cells *via* the uptake of granules or of their exocytosed content by APCs (2). The combination of generation mechanisms favored by early beta-cell dysfunction and of recognition mechanisms favored by T cells that have escaped thymic deletion may endow them with a crucial role in the onset of islet autoimmunity.

Another aspect is the central role that the constitutive proteasome, and its inflammation-induced counterpart, the immunoproteasome, play in the generation of some of these neo-epitopes (39). It is noteworthy that in-vitro exposure of beta cells to interferon (IFN)-γ induces the expression of the immunoproteasome (40) which is known to enhance proteolysis, whereas immunoproteasome-deficient cells accumulate reactive oxygen species (ROS) (41). The immunoproteasome deficiency has also been investigated by in-vivo knockout in mice (42), which led to a CD8+ T-cell-mediated multi-organ autoimmunity comprising insulitis and CD8+ T cells reactive to an IGRP beta-cell peptide with low affinity H-2Kb binding. The authors hypothesized that the constitutive proteasome alone could fail at generating enough high-affinity MHC-I binders. This possibility is supported by other studies highlighting that the immunoproteasome uses the same cleavage sites as the constitutive proteasome, hence impacting the relative quantity rather than the quality of the peptides produced (39, 43, 44). Defective immunoproteasome activity and the resulting decrease in peptide output could thus allow weak-binding immunogenic peptides usually outcompeted by stronger binders to be processed and presented. Conversely, the inflammatory insulitis microenvironment of T1D may induce the well-known MHC-I upregulation (45), but also immunoproteasome expression, thus further enriching the immunopeptidome and favoring strong MHC binders. The higher binding affinity of these peptides may synergize with MHC-I upregulation to increase their availability for T-cell recognition. This increase in beta-cell visibility to T cells may provide one explanation to our recent observation that beta-cell-reactive CD8+ T cells circulate at similar frequencies in T1D and healthy donors (23, 46). This observation suggests that the difference between the ‘benign’ autoimmunity of healthy donors and the progressive autoimmunity of T1D patients may lie not only in their T-cell repertoire, but also in their beta-cell vulnerability (2).

## Pathways for Signal Peptide Epitopes

Insulin is a protein hormone that is initially synthesized as a preprohormone. Preproinsulin is linearly composed of a signal peptide, as most secretory and membrane proteins, a B chain, a C peptide and an A chain (**Figure 2A**). Once the signal peptide is translated, it binds the signal recognition particle that docks to its receptor within the ER membrane and thus allows the direct translocation of newly synthesized insulin into the ER (**Figure 1C**). As the translation continues, the signal peptide is cleaved by signal peptidase (SP) and undergoes intramembrane proteolysis by the signal peptide peptidase (SPP) (47) while the nascent proinsulin is released in the ER. Proinsulin is further cleaved by endopeptidases that remove the C peptide, and folded by chaperones that favor the formation of two disulfide bonds between the B and the A chain, leading to mature insulin stored into granules until its exocytosis. The biosynthesis and secretion of insulin are two distinct and thoroughly regulated processes. Secretion is triggered by a higher glucose concentration (48, 49), meaning that insulin biosynthesis is continually active even at normal glucose concentrations and that most regulation occurs at the secretion stage. This feature has two consequences for APP. First, high numbers of free signal peptides are continually cleaved and released in the cytosol and ER. Second, this high bio-synthetic rate leads to a high number of misfolded insulin molecules, estimated to represent up to 20% of insulin production in normal, unstressed, beta cells (7).

The fate of post-cleavage signal peptides is poorly described. The most studied instance is that of non-classical HLA-E molecules, which present signal-peptide sequences derived from classical HLA-A, -B and -C molecules (50). Surface peptide-loaded HLA-E complexes serve as a marker of proper MHC-I expression and prevent NK-cell activation and killing (51). Even though the presentation of HLA signal sequences by HLA-E is altered when TAP or tapasin are inhibited or knocked-out (52, 53), most reports suggest that the MHC-I presentation of signal peptides is a TAP- and proteasome-independent process (47, 54, 55). The presentation of signal-peptide-derived sequences is also increasingly recognized for classical MHC-I molecules, as described for tumor epitopes (56) and beta-cell peptides (23). We identified several HLA-A2-restricted sequences presented by beta cells and originating from the signal peptide of insulin granule proteins such as insulin, urocortin-3 (UCN3), islet amyloid polypeptide (IAPP) and proconvertase 2 (PCKS2), although the latter was not recognized by CD8+ T cells (23). Interestingly, preproinsulin epitopes restricted for HLA-A2 (23) and other MHC-I variants (57) map to the short (24 aa) signal peptide region. Focused investigations have therefore been conducted to characterize the generation of preproinsulin signal peptides for MHC-I presentation (57, 58). Using K562 cells transfected with a single MHC-I allele and insulin gene, different signal peptide products were found bound to each variant tested, and the fate of insulin signal peptide sequences was dependent on their ER intramembrane location. Indeed, after SPP cleavage within the ER membrane, N-terminal peptides are more likely to be released in the cytosol (**Figure 1C**). These cytosolic free peptides can follow the conventional APP route and be translocated to the ER through TAP transport and subsequently loaded on nascent MHC-I molecules after ERAP1 trimming. On the opposite, upon SPP cleavage, C-terminal peptides are directly released in the ER lumen and loaded on MHC-I molecules, in a TAP- and proteasome-independent manner (57, 58). It is unknown how a peptide can be loaded into MHC-I molecules without TAP and whether ERAP1 trimming is always required, as is the case for a PPI_15-24_ peptide (59). TAPBPR is the chaperone that likely replaces TAP, as tapasin and TAPBPR are mutually exclusive in their binding to MHC-I (55). One of the enzymes suspected to assist peptide trimming in the trans-Golgi network (TGN) is furin. Furin is a proconvertase that is known to traffic from the TGN to the cell surface *via* the vesicular pathway, where it can cleave peptides and proteins (60). Besides being involved in the maturation of secreted proteins such as those of the granule, furin has been shown to process antigens and direct them to the secretory route for MHC-I presentation in a TAP-independent manner (61). Similarly to another proconvertase from the same protein family (PC7) that has been shown to rescue unstable HLA-B51 complexes (62), furin could be crucial in post-ER stabilization of peptide-MHC-I binding. Indeed, resident TGN enzymes like furin could ensure an allele-dependent stability of these complexes against the destabilizing effect of the slightly acidic pH of the TGN. The mono-allelic HLA-I-expressing cells used in these studies may however introduce some bias and might not be representative of (multi-allelic) human beta cells. Consistent with their functional pH range, furin could also be involved in peptide exchanges occurring in the endosomes.

Collectively, the considerable translation rate of insulin and other granule proteins may provide a major source of aAPP pathways already within resting beta cells, through the release of high amounts of signal peptides and further protein processing.

## Concluding Remarks and Future Directions

Beta cells are very active cells with a specialized secretory activity. On one hand, this activity requires unique recycling pathways to maintain homeostasis. On the other, it is a burden that makes beta cells more vulnerable to additional ER stress. These properties may enhance the contribution of MHC-I aAPP pathways in beta cells. Although direct evidence is missing, data from other cellular models make this possibility plausible and invite further investigations. Gaps in knowledge that need to be filled include: 1) the relative contribution of each APP pathway to the beta-cell immunopeptidome under basal and stressed conditions; 2) how these pathways are modulated in the pro-inflammatory environment of insulitis; and 3) whether the same antigenic peptides are generated by professional APCs, a required step to prime naïve T cells in draining lymph nodes before their homing to the pancreas.

## Author Contributions

AC and RM wrote the manuscript. All authors contributed to the article and approved the submitted version.

## Funding

Work in our Laboratory is supported by The Leona M. and Harry B. Helmsley Charitable Trust (1901–03689), JDRF (2-SRA-2016-164-Q-R), the *Agence Nationale de la Recherche* (ANR-19-CE15-0014-01), the *Fondation pour la Recherche Medicale* (EQU20193007831), and the Innovative Medicines Initiative 2 Joint Undertaking under grant agreements 115797 and 945268 (INNODIA and INNODIA HARVEST), which receive support from the EU Horizon 2020 program, the European Federation of Pharmaceutical Industries and Associations, JDRF, and The Leona M. & Harry B. Helmsley Charitable Trust.

## Conflict of Interest

The authors declare that the research was conducted in the absence of any commercial or financial relationships that could be construed as a potential conflict of interest.
